# Married to the Pacemaker: An Autobiographical Case Report of Sick Sinus Syndrome

**DOI:** 10.7759/cureus.58017

**Published:** 2024-04-11

**Authors:** Shafee M Khan

**Affiliations:** 1 Respiratory Medicine, Datta Meghe Medical College, Datta Meghe Institute of Higher Education and Research, Deemed to be University, Nagpur, IND

**Keywords:** sinus node dysfunction, complete heart block, pacemaker, autobiographical case report, sick sinus syndrome

## Abstract

I, the author of this case report, was on beta blockers, initially atenolol and later on nebivolol, for my "systemic hypertension" illness. After attending the National Pulmonary Conference, I fell during the return journey on the express highway, became unconscious, and reached the tertiary care hospital of the medical college at the end of the day. The electrocardiogram was suggestive of a "complete heart block.". They immediately implanted a temporary pacemaker and transferred me to the cardiac care unit. I was discharged after five days of stay in the hospital, as the Holter study concluded to be normal. After about two weeks, I felt lightheadedness and giddiness for a fraction of a second. An eminent senior cardiologist in my hometown advised Holter's study; this time, it was suggestive of long pauses. A permanent pacemaker was implanted with the diagnosis of sick sinus syndrome. This autobiographical case report hopes to shed light on more advanced cardiac screening in the search for the etiology of clinical symptoms.

## Introduction

Sinus node dysfunction (SND) or sick sinus syndrome (SSS) is common due to age-related changes (fibrosis and sclerosis) in the conduction system of the heart. SND encompasses a range of sino-atrial node abnormalities. The likely diagnosis is due to variable manifestations on the electrocardiogram (ECG): junctional bradycardia, asystole, atrioventricular block (AV block), and atrial tachycardia syndrome [1]. Even though atrioventricular blocks are more common, a complete heart block is comparatively uncommon [2]. In the general population, it is found to be as low as about 0.02 to 0.04% [3]. In evidently healthy and most likely asymptomatic people, it is as low as 0.001% [4]. Among the diseased population, as per the research findings of the individuals in the Veterans Health Administration, it was noticed to the extent of 1.1% in diabetic individuals and 0.06% in hypertensive patients [5]. Patients with sino-atrial node dysfunction may be asymptomatic or present with lightheadedness, dizziness, confusion, and syncope due to a transient decrease in cardiac output. Tonic or clonic seizures may also occur if there is a prolonged period of cerebral hypoperfusion. However, this is rare; only a few cases have been reported.

## Case presentation

Presenting complaints 

I traveled with my senior colleague for three days from my office in Central India to attend the National Pulmonary Conference in the famous city of North India, Agra (home of the Taj Mahal). We stopped for lunch at a restaurant in the middle of our return journey after the conference was over in the afternoon, and as soon as I took the plate in my hand, I fell to the ground and became unconscious. After requesting an ambulance, we traveled on the express highway to the health center. In the meantime, I started to feel better. After receiving primary medical assistance from the paramedical staff of the health center, we continued our journey. We arrived at the emergency room of the tertiary care hospital of the medical college in the capital city of India, Delhi, in the evening. A cardiology department resident on duty assessed the situation; an electrocardiogram (ECG) was conducted, suggesting a complete heart block. I was immediately transferred to the hospital's cardiac catheterization laboratory, where a temporary pacemaker was implanted through the left femoral vein. Then, I was transferred to the cardiac care unit (CCU) for monitoring, additional tests, and observation. 

History 

I had hypertension and used a B-blocker for five years; initially, I took atenolol 25 mg once a day. I later consulted with the physician at my workplace. Following a preliminary assessment, the physician prescribed a newer generation B-blocker, nebivolol (5 milligrams once a day), for my "systemic hypertension" condition. In the follow-up examination conducted fifteen days later, he stated that this newer generation B-Blocker produced the best blood pressure management. This made me extremely happy.

I used to work out by taking quick morning walks that lasted about an hour. After about three months of physician consultation, I traveled with my senior colleague to attend the National Pulmonary Conference in the far-off city of North India described above, which is roughly 1000 kilometers from my town. We stopped for lunch in a restaurant on an express highway after the conference ended as we traveled back by car, and as soon as I stood up to take a grip of the food plate, I fell to the ground and became unconscious. An ambulance was called, and I was transported to the express highway health center. Meanwhile, I regained consciousness, and after receiving primary health care, we arrived at the tertiary care hospital of the medical college in Delhi, the Indian capital city. In the emergency room, I was clinically evaluated by the cardiology department resident. A few basic vital parameters were noted. An electrocardiogram (ECG) was requested and revealed a complete heart block. Immediately, I was brought to the cardiac catheterization laboratory, where temporary pacemaker implantation (TPI) was completed through left femoral venous access and shifted to the CCU for further observation and monitoring.

In the CCU, a Holter recorder was used to investigate irregularities in cardiac rhythm, and other vital parameters were continuously monitored. After five days of stay in the CCU, I was discharged with the diagnosis "hypertension with complete heart block (transient), reverted to sinus rhythm spontaneously? drug-induced." To treat hypertension, the B-blocker nebivolol was substituted with the angiotensin receptor blocker telmisartan, 40 milligrams once a day, with the warning not to take a B-blocker once more. Remarkably, the results of the Holter examination and other basic investigations were normal.

**Figure 1:**
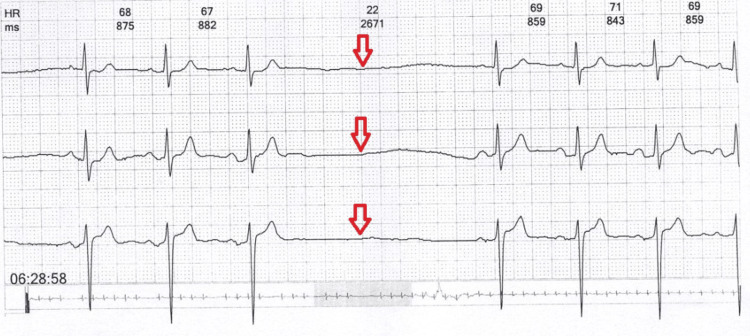
A Holter study with electrocardiography revealed long sinus pauses (red arrows) that suggest a complete heart block.

After a fortnight of being back at work, I had shortness of breath, giddiness, and lightheadedness after sitting in my pulmonary outpatient department room. I consulted with a cardiologist in my town, who suggested a Holter study and other basic investigations. This time, a Holter analysis indicated that there were long sinus pauses (Figure 1). Another eminent cardiac electrophysiologist who visited my hometown regularly evaluated me. After a thorough evaluation, the cardiologists recommended I get a permanent pacemaker implantation (PPI).

**Figure 2:**
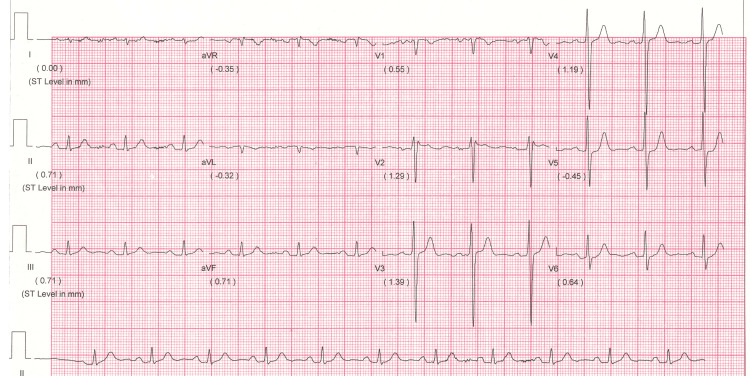
An electrocardiogram done after permanent pacemaker implantation (PPI) showed normal sinus rhythm with no evidence of complete heart block.

Finally, at the renowned cardiac hospital in my town, a permanent pacemaker (Dual Chamber DDDR, MRI compatible) was implanted through the left venous approach within my heart. After three days, the hospital discharged me with a diagnosis of "sick sinus syndrome" and stable hemodynamics, noting that I was permanently married to a cardiac pacemaker, requiring proper care and adequate follow-up. I have followed up regularly with the local cardiologist for five years. The post-pacemaker insertion Holter study was within normal limits (Figure 2).

## Discussion

Two eminent senior cardiologists evaluated and advised permanent pacemaker implantation for my ailment, depicting the strength of the management. More advanced investigations could not be done as they were unavailable at that time, pointing to its weakness. Sinus node dysfunction (SND), or sick sinus syndrome (SSS), is a condition of the sino-atrial node (SA node) brought on by a constellation of aberrant rhythms due to reduced pacemaker performance and impulse transmission. Some of the abnormal rhythms are bradyarrhythmias, tachyarrhythmias, and tachy-brady syndrome, which is tachycardia alternating with bradycardia. These arrhythmias may cause tissue hypoperfusion, tiredness, palpitation, dizziness, presyncope, and syncope. The etiological factors for SND may be classified as intrinsic and extrinsic. Intrinsic damage is caused mostly by the fibrosis of the tissue of the sino-atrial node itself, and extrinsic reasons influence the nodal function. SND can come from congenital disorders, arrhythmia, infiltrative disorders, or surgery; by and large, congenital abnormalities are the most frequent intrinsic factors, and age-specific sinus node degeneration is the most common intrinsic cause of SND [6].

In animal models, it has been demonstrated that heart failure and tachyarrhythmias cause the sinus node to undergo cellular remodeling [7]. The sino-atrial node may be invaded by collagen vascular disease, sarcoidosis, amyloidosis, or metastasis due to malignancy and may cause SND. Injury to the sino-atrial node or the artery feeding it may occur after performing cardiac operations, such as replacement of heart valves, transplant surgery of the heart, procedures for correcting congenital heart defects, and, thus, SND. Infectious diseases like bacterial endocarditis and Chagas disease are less prone to SND than the issues of atrioventricular conduction. It is unusual for atherosclerosis of the arteries supplying the SA node to induce ischemic injury since the SA node resides inside the atrial wall [8].

Numerous outside factors can influence how well the SA node paces. These can appear in situations such as hypersensitivity of the carotid sinus, vasovagal syncope, and autonomic dysfunction with excessively elevated vagal tone. Additionally, metabolic abnormalities like hypothyroidism, hyperkalemia, hypokalemia, hypocalcemia, hypoxia, and hypothermia might impair the pacing ability of the heart's sinus node. Obstructive sleep apnea can induce bradycardia by causing severe hypoxia during episodes of apnea. Bradycardia can also result from increased intracranial pressure (Cushing's reaction). Class I to IV antiarrhythmic drugs, digoxin, lithium, and sympatholytic drugs are a few pharmacologic and toxic agents that can similarly impact the SA node.

Although it can happen at any age, SND is an ailment primarily affecting elderly people. One of the 600 heart disease patients, 65 years of age or older, will have SND. Both men and women are equally impacted. SND was the only cause in the year 1990 in the United States for more than 50% of pacemaker implantation [9]. A complete AV block can occur yearly, ranging from 0% to 4.5%, with a median of 0.6% [10].

The evolution of SND, naturally, typically takes years. In most individuals, no symptoms are noticed initially. Less oxygen delivery to the vital organs is frequently responsible for the occurrence of the symptoms in these patients. The central nervous system is under-perfused due to sinus pauses or severe bradycardia, which can cause paroxysmal presyncope or syncope. Syncope can also occur when the abrupt cessation of atrial fibrillation (AF) in tachy-brady syndrome leads to a protracted sinus halt and incompetency to restore normal sinus rhythm. There could be renal and gastrointestinal hypoperfusion, causing oliguria and discomfort in the abdomen, respectively. The prognosis is poorest for rhythms that include AF, or atrial flutter because they significantly increase the chance of thromboembolism, which can result in a transient ischemic attack or cardioembolic stroke. Fatigue and exercise intolerance are the results of chronotropic incompetence. Palpitations are typically experienced during tachycardia bouts, following the cessation of tachyarrhythmias, or as a result of lengthy pauses. Additionally noted are worsening angina pectoris or congestive heart failure.

For the evaluation of SND, initially, the reversible abnormalities like electrolyte imbalance, obstructive sleep apnea, and metabolic disorder, as well as the offending drugs responsible for the suppression of the sinus nodal tissue like B-blockers and calcium channel blockers, must be excluded. The symptomatology of the patient should correspond with the bradycardiac event and be recorded. As the condition of SND is episodic, the diagnosis depending on the findings of a traditional electrocardiogram is less likely; hence, surveillance for a long duration is recommended to identify abnormal arrhythmic events. Either a 24-48 hours Holter monitor or telemonitoring is advised for this purpose. If telemetry or Holter study is non-conclusive, then a long-term event monitor, external or implantable, a loop recorder, or both are essential to diagnose SND.

After carotid sinus massage, a prolonged sinus pause of more than three seconds is not solely diagnostic but suggests SND. If there is a high suspicion of SSS and no arrhythmic episodes are noticed, then an electrophysiologic study is recommended. The treadmill exercise test is most commonly used for diagnosing chronotropic incompetence, specifically in people with fatigue and symptoms after exertional activities. Although this has not been clinically verified [11], the diagnosis of chronotropic incompetence is made when the maximal expected heart rate attained by the patient is less than 80%. 

Reversible variables must be found and addressed as a first step in treating SND. After addressing reversible causes, a permanent pacemaker may be installed to treat SND. A permanent pacemaker is considered for patients with proven bradycardia as the cause of their symptoms and chronotropic incompetence. A permanent pacemaker is also recommended for drug-induced (required for other medical conditions) SND. In tachy-brady syndrome, when AF is treated with B-blockers, the SA node is suppressed, leading to extended, long pauses and syncope when an episode of tachyarrhythmia halts. Furthermore, it is reasonable to consider implanting a permanent pacemaker in symptomatic individuals with fewer than 40 beats per minute bradycardia that was not previously connected with their symptoms. It makes sense for patients with diagnostic electrophysiologic testing to get a permanent pacemaker implanted. Patients with a persistent third-degree AV block are advised to get a pacemaker, albeit the phrase "persistent" is frequently a matter of clinical discretion [11]. In an Italian study, 21% of patients with little over 24000 participants underwent pacing for third-degree AV blockages [12]. Due to the higher risk of AV block in SND, dual-chamber pacing is typically chosen when choosing a pacemaker type. Since individuals with paroxysmal atrial tachyarrhythmias have a higher risk of stroke, anticoagulation should be addressed in these patients. Due to a higher risk of AV block, dual-chamber pacemakers are advised for patients with sinus SND. Furthermore, compared to individuals who solely receive ventricular pacing, atrial pacing achieved with a dual-chamber pacemaker may lower the incidence of AF and subsequent risk of stroke and systemic thromboembolism [13]. Importantly, SND has been proven not to affect mortality whether treated with a pacemaker or not, and it progresses benignly with minimal risk of sudden cardiac death [14-16].

## Conclusions

Before prescribing anti-hypertensive medications, the case of systemic hypertension should be carefully assessed, and all pertinent advanced investigations should be sought, taking into account any detrimental effects on cardiac tissue. One should request Holter monitoring, echocardiography, telemonitoring, and loop study if necessary before prescribing an anti-hypertensive drug, which is known to have a depressive effect on the conducting tissues of the heart and may result in a syncopal episode. The most significant factor contributing to transient loss of consciousness is cardiac origin syncope. For the majority of people, the diagnosis is difficult. To successfully manage the patient and avoid the pharmacological agents responsible for exacerbating the preexisting disease and causing loss of consciousness, which may be catastrophic and may result in death, it is imperative to have an accurate diagnosis. This is accomplished by exposing the patient to pertinent investigations and interventions.
